# Development and evaluation of a training model for paracentetic suprapubic cystostomy and catheterization

**DOI:** 10.6061/clinics/2019/e435

**Published:** 2019-04-10

**Authors:** Wei Gao, Tongwen Ou, Jianguo Jia, Jie Fan, Jianjun Xu, Jin Li, Xin Cui, Xinzhou He, Xueli Li

**Affiliations:** I Department of Urology, Xuanwu Hospital, Capital Medical University, Beijing, China; II Department of Education, Xuanwu Hospital, Capital Medical University, Beijing, China; III IMS Market Research Consulting (Shanghai) Co., Ltd. Beijing Branch, IMS Health, China

**Keywords:** Suprapubic, Cystostomy, Puncture, Training Model

## Abstract

**OBJECTIVES:**

Minimally invasive paracentetic suprapubic cystostomy is a technique that should be learned by all surgical trainees and residents. This study aimed to develop a self-made training model for paracentetic suprapubic cystostomy and placement of the suprapubic catheter and then to evaluate its effectiveness in training fourth-year medical students.

**METHODS:**

Medical students were divided into an experimental group receiving comprehensive training involving literature, video, and model use and a control group receiving all the same training protocols as the experimental group except without hands-on practice using the model. Each student's performance was video-recorded, followed by subjective and objective evaluations by urology experts and statistical analysis.

**RESULTS:**

All students completed the surgical procedures successfully. The experimental group's performance scores were significantly higher than those of the control group (median final performance scores of 91.0 *vs*. 86.8, respectively). Excellent scores were achieved by more students in the experimental group than in the control group (55% *vs*. 20%), and fewer poor scores were observed in the experimental group than in the control group (5% *vs*. 30%).

**CONCLUSIONS:**

Based on its cost-effectiveness, reusability, and training effectiveness, this paracentetic suprapubic cystostomy training model is able to achieve goals in teaching practice quickly and easily. Use of the model should be encouraged for training senior medical students and resident physicians who may be expected to perform emergent suprapubic catheter insertion at some time.

## INTRODUCTION

Suprapubic cystostomy, also known as vesicostomy or epicystostomy, is a surgically constructed connection through the abdominal wall into the urinary bladder to allow drainage of urine from the bladder when normal urinary flow is restricted. Suprapubic cystostomy is a basic surgical procedure in urology that can be performed as an open suprapubic cystostomy or minimally invasive suprapubic cystostomy. Development of the latter has resulted in the open approach being rarely used 1. Suprapubic catheter insertion is widely used to manage acute or chronic urinary retention 2 and neurological diseases (e.g., multiple sclerosis and spinal cord injury) 3, as well as its being common used for urinary incontinence, urethral trauma, and the simplified care of weakened elderly patients 4. The procedure may also be performed during investigative or surgical procedures or for postoperative bladder drainage 4, including with cystoscopy, ultrasound-assisted positioning, and auxiliary transurethral catheterization 5,6. Complications associated with transurethral catheterization (e.g., urethritis, epididymitis, or stricture) can be avoided with suprapubic paracentetic cystostomy 7. However, risk of complications may increase with incorrect performance of the technique or with poor training and education 8. The incidence of bowel perforation or other injury is reported to be as high as 2.7%, and iatrogenic direct rectal injury has also been reported 9.

Given the widespread use of suprapubic catheter insertion and the reported risk of complications if performed incorrectly, undergraduate medical students without clinical experience, senior medical students, and urology and surgery residents with standardized training would benefit from learning this technique. However, until recently, the conventional teaching mode for suprapubic cystostomy has only allowed students to watch a video of a suprapubic puncture and catheter insertion and then to practice on a patient under the guidance of an experienced surgeon (opportunity training). The standard teaching tool available commercially is a simulated bladder made of silicone placed in an abdomen. Although this tool is relatively expensive, it is good for only one puncture, cannot be reused and is not conducive to teaching and simulating the technique for medical students.

An appropriate training model should have the following qualities: it must mimic the real clinical environment and simulate the entire surgical procedure; it should provide accurate sensory feedback (i.e., felt by the surgeon's hands); multiple copies of the model must be available for rapid cycling through simulations to ensure that all trainees are able to practice the technique; components of the model should be inexpensive and easy to replace, with a low overall cost for the entire model; and, most importantly, the model should be conducive to improving trainee understanding of the procedures and increasing their confidence in performing the operation 5,6. Among many studies introducing training models for suprapubic cystostomy 5-7,10-12, the above guidelines were applied in two studies introducing cost-effective simulation training models 5,6. Shergill et al. 5 developed and introduced a safe, simple model of suprapubic catheter insertion to junior doctors in a urology emergency training program, thus improving their ability to perform the procedure in clinical practice with repetitive use. Hossack et al. 6 developed a realistic model with sensory feedback, one that could be replicated easily in any clinical training facility and could be used to train students effectively. However, the shortcomings of these models include the relatively high cost of production and the cumbersome process involved in the reuse of the model. We used these two previously developed training models as a basis for a modified design and developed a simple, practical, reproducible and cost-effective training model. We sought to evaluate the model's use in training pre-clinical medical students without prior patient contact. The purpose of this study was to develop a self-made teaching model for suprapubic catheter insertion in urological paracentetic suprapubic cystostomy and placement of the suprapubic catheter and to evaluate its effectiveness in training fourth-year medical students.

## MATERIALS AND METHODS

### Study design and subject recruitment

This prospective, observational study was conducted between January and December 2014 to develop and test a training model for suprapubic cystostomy. A total of 40 fourth-year medical students were recruited from the medical school enrollment by convenience sampling. Each participating student provided written informed consent. The students were randomly divided into two groups, an experimental group and a control group, each comprising 20 students. A review panel of six urologists (everyone had more than 25 years of experience) was invited to participate in judging the students' performance with the developed model.

### Materials for construction of the training model

The main materials used in constructing the model included a plastic microwave food container (LOCK&LOCK TRADING Co., Ltd., Shanghai, China), medical sterile latex gloves (RuiJing latex products Co. Ltd., Beijing China), Styrofoam filler (China HP Co. Ltd., Beijing, China), and a medical adhesive bandage (Idealast-haft, PAUL HARTMANN AG, Heidenheim, Germany) for orthopedics. A Urovision® bladder puncture kit (Sanlin International, Beijing, China) with 14 French (F14) puncture needle, (uroVision GmbH, Bad Aibling, Germany), F14 Frye's double lumen urinary catheter (uroVision GmbH), trocar with handle (uroVision GmbH) and an obturator at the catheter end (uroVision GmbH), as well as a 5-ml syringe (Shandong Weigao Group Medical Polymer Company Limited, Co. Ltd, China), syringe holder (Shanghai Medical Instruments (Group) Ltd., Corp, China), toothed forceps (Shanghai Medical Instruments (Group)), angular needle (JinHuan Medical Products Co. Ltd., Shanghai, China), and silk thread (JinHuan Medical Products Co. Ltd.,) were used to simulate the surgical procedure.

### Methods for model construction

**Figure 1:**
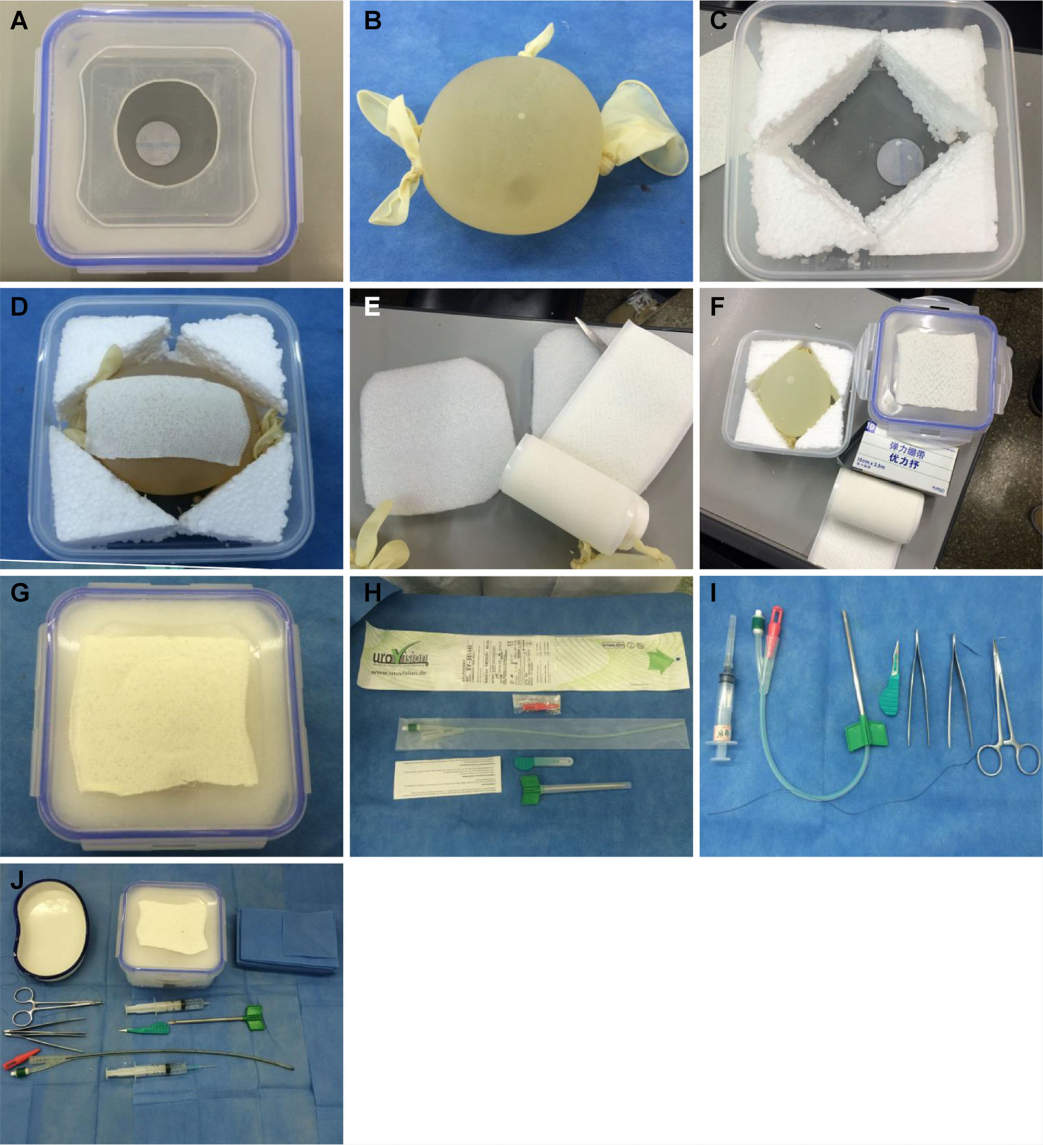
All equipment used. Figure 1A. A circular hole with a 6-cm diameter is created in the center of the microwave food container lid. Figure 1B. Large, sterile latex glove filled with sterilized water + iodine, fastening five fingers and cuff with rubber bands to create a spherical water bladder. Figure 1C. Styrofoam is used to fill the surrounding space in the microwave food container. Figure 1D. Water bladder is placed in the middle of the microwave container and covered with adhesive bandage to increase puncture resistance and prevent bursting. Figure 1E. Top of the water bladder is covered with Styrofoam to simulate the uppermost layer of the abdominal wall. Figure 1F. Surface of the microwave container is covered with adhesive bandage to simulate the skin surface. Figure 1G. Microwave container is closed tightly to form a simulation model for suprapubic paracentetic cystostomy. Figure 1H. Obturator at the urinary catheter end is used to close the end of the urinary catheter to allow monitoring of the effluent. Figure 1I. A 5-ml syringe simulates local anesthesia to the skin and is used to inject water into the bladder through the urinary catheter for fixation. Figure 1J. Needle holder, toothed forceps, angular needle, and silk thread are used to suture and fix the urinary catheter.

A circular hole with a 6-cm diameter was created in the center of the lid of the microwave food container (Figure 1A). A large, sterile medical latex glove was filled with sterilized water containing iodine. The five fingers and the cuff of the glove were fastened to make a spherical water bladder (Figure 1B). Styrofoam was used to fill the surrounding space in the microwave food container (Figure 1C). The water bladder was placed in the middle of the microwave container and covered with an orthopedic medical adhesive bandage to increase the puncture resistance and prevent the water bladder from bursting (Figure 1D). The top of the water bladder was covered with Styrofoam to simulate the uppermost layer of the abdominal wall (Figure 1E). The surface of the microwave container was covered with the adhesive bandage to simulate the skin surface (Figure 1F). The microwave container was tightly closed with the lid to form the simulation model for paracentetic cystostomy (Figure 1G).

To simulate the surgical procedure, the Urovision® bladder puncture kit was used as follows. The F14 puncture needle was connected with an F14 Frye's double lumen urinary catheter to puncture the water bladder and insert the urinary catheter. The trocar was used to cut the skin and subcutaneous tissue. The obturator at the urinary catheter end was used to close the end of the urinary catheter before starting so that the effluent could be monitored (Figure 1H). A 5-ml syringe was used to simulate local anesthesia of the skin and inject water into the bladder through a urinary catheter for fixation. The needle holder, toothed forceps, angular needle, and silk thread were used to suture and fix the urinary catheter (Figure 1I). All equipment used is shown in Figure 1J.

### Paracentetic cystostomy procedure

The paracentetic cystostomy model was placed in the center of an operating table, with routine disinfection by iodine alcohol and draping. A 5-ml syringe was used to simulate local anesthesia on the skin surface, and the center of the model was selected as the site for suprapubic incision. A trocar was used to cut the skin, subcutaneous tissue and front sheath with a 1-cm long incision (skin+subcutaneous+front sheath). The catheter was nested into the puncture needle but did not reach the tip of the needle, which was rotated vertically downward along the incision. When the breakthrough was felt, the needle was inserted an additional 1 cm (approximately). The urine outflow was checked (at the breakthrough point and then after the needle entered an additional 1 cm). The catheter was placed along the puncture needle to allow the bifurcated end of the catheter to reach the rear end of the puncture needle (preset in advance). Through the catheter, 5 ml of water was injected into the water bladder. Together with the connected puncture needle, extubation was performed upward. When resistance was sensed, the water bladder was fixed at the edge of the simulated bladder. Urine drainage was checked again. Two sutures were performed on the simulated skin surface to fix the catheter. After the puncture operation, the microwave container was opened to check the completeness and tightness of the bladder model. The catheter was removed to check urine spraying at the puncture site and signs of puncture in the simulated bladder.

### Demonstration of a self-composite model of suprapubic paracentetic cystostomy

**Figure 2:**
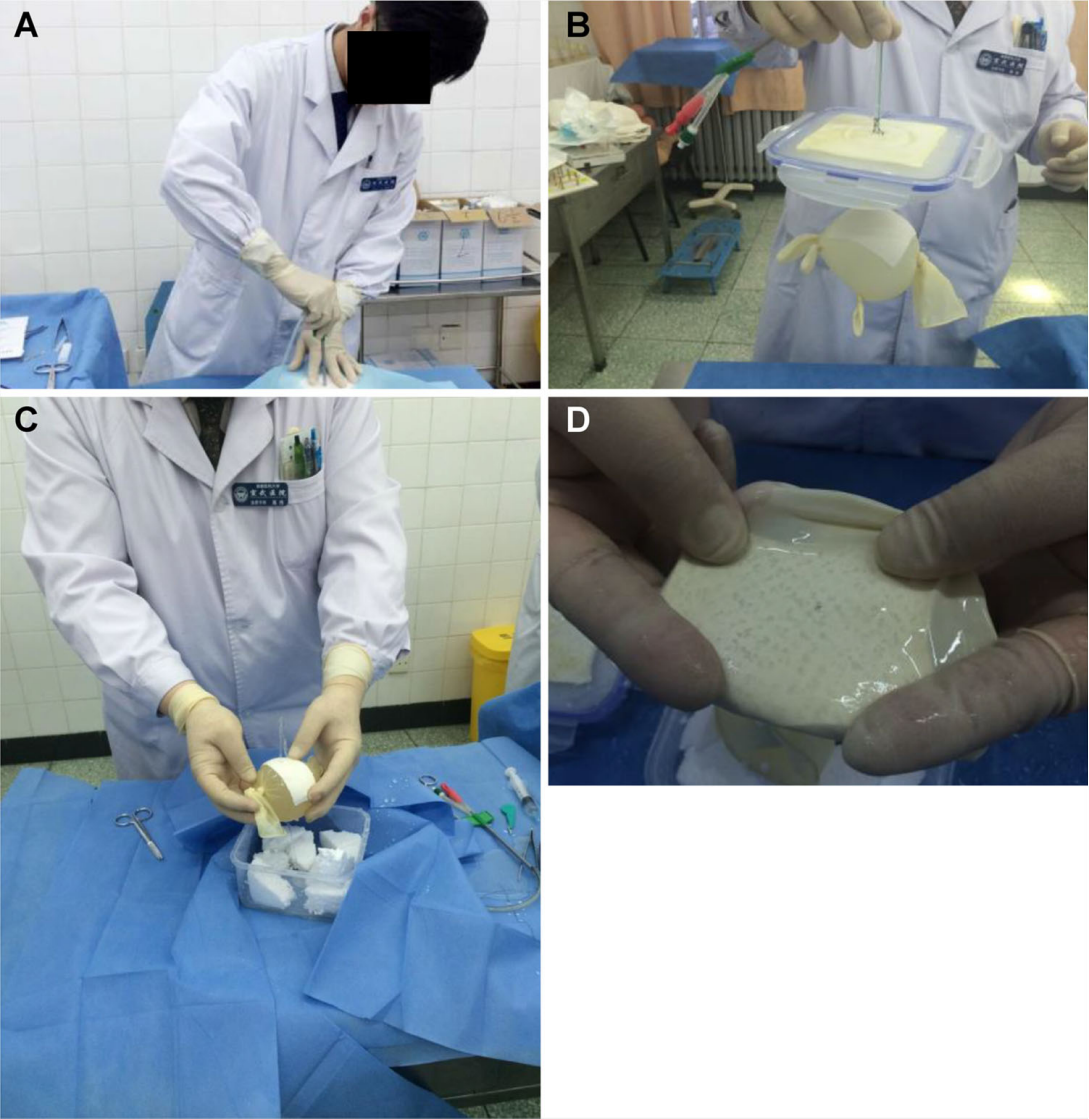
Demonstration of self-made training model for suprapubic paracentetic cystostomy. Figure 2A. Suprapubic paracentetic cystostomy simulation performed by a student. Figure 2B. After puncture, the tightness of the connection between the catheter and simulated bladder is checked to rule out extravasation. Figure 2C. After puncture, the catheter is removed to observe water flow from the simulated bladder. Figure 2D. Glove used as the simulated bladder is extracted to observe the puncture hole and signs of puncture.

In Figure 2A, a student is performing the paracentetic cystostomy simulation. After the puncture, the tightness of the connection between the catheter and the simulated bladder was checked to ensure that no extravasation had occurred (Figure 2B). After the puncture, the catheter was removed to observe the water flow of the simulated bladder (Figure 2C). Then, the glove, or simulated bladder, was extracted to observe the puncture hole and signs of puncture (Figure 2D). The mean water amount needed to maintain the shape of the artificial bladder was 494.93±19.97 ml with a range from 435 ml to 532 ml (measurements repeated 5 times). The mean water volume was calculated by puncturing the artificial bladder (sterile glove) and releasing the water into a measuring cylinder. Although the gloves are the same size, including consistent diameters, differences in water volume may occur between simulated bladders without affecting model performance.

### Training process and evaluation of its effectiveness

The trainees were fourth-year medical students who had not yet entered the clinical practice stage and had no prior patient contact. Thus, they had not received any training in clinical practice and had no prior experience with suprapubic catheter insertion or the associated bladder breakthrough during the procedure. The students were randomly divided into two groups, an experimental group and a control group, each comprising 20 students. Students in the experimental group were asked to read the English literature related to paracentetic cystostomy and modeling in advance (the reading period was the entire winter break for approximately 50 days) 13-18. These students watched the instructor's video of a suprapubic catheter insertion before they personally participated in preparing the suprapubic paracentetic cystostomy model and practiced the puncture and catheter insertion with the model. The students in the control group had the same training protocol as the experimental group, including reviewing related English literature and watching the instructor's video of suprapubic catheter insertion, but they had no hands-on practice in preparing the suprapubic paracentetic cystostomy model and no practice with the puncture and catheter insertion using the model 13-18.

The main outcome measure was performance. The performance of all students was recorded on video, saved, and then scrambled randomly between experimental and control group students for viewing by urologists. The design of the operating procedure involved ten main steps, and each step was evaluated by a group of five urologists from our hospital to assign a weighted value. The urologists then scored the puncture operation and catheter insertion of each student by watching the video of that student's performance, resulting in a subjective score, which was weighted as 60% of the final score. The time spent on each step by a student formed an objective score, which was obtained by an evaluation of each student's performance in the video. Compared to the standard time for each step, the specific score for each step was determined for each student. The weight of each step is equivalent to that of the subjective score. If the time exceeded the standard by 10 seconds in a given step, one point was subtracted; differences relative to the standard of less than 10 seconds were treated as a difference of 10 seconds. For only the suture step, one point was subtracted for exceeding the standard by 60 seconds. The standard time in each step was determined according to the operating time of the chief urologist, who is the first author of the current paper, among a total of five urologists. The objective score was weighted as 40% of the final score. The sum of the total subjective score and the total objective score was recorded as the final score for each student and was retained for statistical analysis.

In accordance with the pre-designed scoring criteria, six urologists, each with more than 25 years of experience, carefully watched each student's video and assigned a subjective score to each video. Next, the objective score was determined for the same student based on the time spent on each step in the video. The total subjective score (60%)×0.6 plus the total objective score (40%)×0.4 (the weighting of 0.6 and 0.4 were co-determined by the six urologists) resulted in the student's final score and was retained for statistical analysis.

### Statistical analysis

Continuous variables, such as total scores, are presented as medians and inter-quartile ranges, and a Mann-Whitney U test was used for group comparison. Categorical variables are presented as counts and percentages, with Fisher's exact tests used for group comparison.

Statistical analyses were performed with IBM SPSS statistical software version 22 for Windows (IBM Corp., Armonk, New York, USA). A two-tailed *P* < 0.05 indicates statistical significance.

## RESULTS

### Subjects' baseline characteristics

A total of 40 subjects were enrolled in this study and were randomized into the following two groups: 20 subjects in an experimental group and 20 in a control group. Each group included 6 males and 14 females. According to the medical school system in China, the academic programs are classified as seven-year medical school programs (starting from high school graduates) and five-year medical school programs (starting from college graduates). The experimental groups and control groups included 16 and 14 students from 7-year medical schools, respectively, and 4 and 6 students from 5- year medical schools, respectively. No statistically significant differences were found in the baseline characteristics between experimental group and control group students from the two academic medical school programs (*p*=0.761).

### Comparisons of performance scores between experimental and control groups

An exemplary list of objective scores of students' performance is shown in Table 1. Table 2 shows a sample list of subjective scores assigned by the urologists who watched the videos of the students' performance and assigned the subjective scores. Table 3 shows a list of the raw scores of all students by group and gender (equal numbers of males and females in each group).

The median final score was significantly higher in the experimental group than in the control group (91.0 *vs*. 86.8, *p*=0.002). The final scores used by six urologists to describe student performance were graded as poor, good, or excellent. The performance levels correlated significantly with the two groups (*p*=0.035). Excellent scores were achieved by more students in the experimental group than in the control group (55% *vs*. 20%), and fewer poor scores were observed in the experimental group than in the control group (5% *vs*. 30%) (Table 4).

### Cost-effectiveness

The total cost of producing our model is RMB 109. Without including the catheter set, which is a separate expense, the cost would be less than $2.00 US. Two sets of catheters purchased for training purposes cost RMB 1070 in total, which is still a comparable or lower cost than that of similar models 6.

## DISCUSSION

In this study, a simple, practical training model for suprapubic cystostomy was designed using readily accessible materials, including a plastic microwave food container, a medical sterile glove filled with water to simulate the bladder, packaging Styrofoam to simulate the structure of the abdominal wall, and a medical elastic adhesive bandage to simulate the abdominal skin and rectus sheath. Multiple tests confirmed that this simple model simulated the entire procedure of suprapubic cystostomy. The model is simple, inexpensive, and reusable and can be reproduced quickly and easily in any medical training facility. Most importantly, the results of the present study showed that all the medical students using the model could complete the procedure successfully. However, differences were observed between the experimental group that received comprehensive training with an extensive literature review, instruction video, and model use and the control group that only watched a video instruction before model use. When graded by six urologists who watched video recordings of each student's performance, the experimental group performance scores were significantly higher than those of the control group (median final performance scores of 91.0 *vs*. 86.8, respectively). Excellent scores were achieved by more students in the experimental group than in the control group (55% *vs*. 20%), and fewer poor scores were observed in the experimental group than in the control group (5% *vs*. 30%).

The entire puncture process was conducted using a Urovision® bladder puncture kit. The only significant difference between our process and the standard suprapubic cystostomy is that we placed the catheter along the puncture needle in advance so that the puncture and catheterization procedure could be completed in one step. This process increased the ease and reliability of the procedure, and this one-step approach may contribute to the wider application of suprapubic catheters, as demonstrated in a previous study 7. The Urovision kit and all procedures we used are almost identical to those proposed by Reuter and Raible 1 in 2014. However, in our model, after the puncture was completed, the puncture needle was not destroyed, and the puncture needle and the catheter were reused. Other techniques have been incorporated into training models. Mohammed et al. 10 applied the Seldinger technique for paracentetic cystostomy using the MediPlus suprapubic catheter kit, which was shown to provide safe introduction of the catheter into the bladder using a variation of the traditional technique. In comparison to these traditional methods, our model again eliminated the step of catheter placement after removing the puncture needle core, by placing the catheter along the puncture needle in advance. Both experienced practitioners and trainees expressed satisfaction with using an anatomically correct model developed by Singal et al. 11. The main features of that model were its low cost and easy reproducibility, with only bladder, skin, and fat layers needing periodic replacement. These features parallel our interest in developing an economical, reproducible model. However, the method of Reuter and Raible 1 still compares most favorably with that applied in our clinical practice.

The purpose of developing our model was to help fourth-year medical students understand and memorize all aspects of the suprapubic cystostomy procedure, a clinical training that would prepare them for clinical practice. We also wanted them to have the experience of the “breakthrough” that occurs during the puncture operation; this breakthrough had not been experienced in any previous procedural training. By watching the video of the operation recorded by the instructor for all the students, an obvious pause during the puncture on the simulated bladder was observed. At this point, the puncture needle was inserted approximately 1 cm more, and catheterization was performed after checking the urine outflow from the end of the catheter. The results of the students' questionnaires showed that all students had successfully completed the catheter insertion procedures and claimed that they experienced the sense of breakthrough. Among all students, 87.5% declared that in a future practice stage, they would be confident in their ability to successfully perform this puncture operation for patients requiring paracentetic cystostomy. As Manalo et al. 19 concluded in their study of urethral catheter insertion by medical interns, thorough training is absolutely necessary to reduce the risk of complications and injury. Those authors recommended universal training given at an appropriate time during the medical students' curriculum.

### Limitations

The results of this study are limited by the fact that it was conducted in a single center. Experience in only a single institution may introduce institutional bias. In addition, convenience sampling was used to select 40 student participants, which does not rule out sampling error and selection bias. The use of a large, sterile medical latex glove filled with sterilized water containing iodine to mimic a spherical water bladder is somewhat primitive. The materials and construction of the current model need further testing to better simulate the performance of suprapubic cystostomy in clinical practice. Further cooperation with medical model manufacturers as well as sharing our basic design may help to produce a professional, realistic model for superior simulation training. Finally, whether this model subsequently translates into improved clinical performance is not addressed. Since Chinese law prohibits students from actually placing suprapubic catheters in patients, we cannot address the true patient outcome questions, such as measurements of confidence performing the actual procedure, success of catheter placement, and minimization of complications. This study was an initial observational evaluation by urology experts of the effectiveness of our model for training fourth-year medical students, but a validated scoring system is needed. Our future studies will incorporate validated scoring methods, such as Objective Structured Assessment of Technical Skills (OSATS) scoring, for more reliable and valid surgical skills assessment.

In conclusion, the paracentetic suprapubic cystostomy training model developed and evaluated in the present study is simple, practical, inexpensive, and reusable and is reproduced easily and quickly to achieve training goals in teaching practice. Trainees readily become familiar with and understand bladder puncture procedures, which may help to increase their confidence when they perform suprapubic catheter insertion in clinical practice. The training model is appropriate for the training of senior medical students and resident physicians who may be expected to perform emergent suprapubic catheter insertion at some time.

## AUTHOR CONTRIBUTIONS

Gao W, Ou T, Fan J, Xu J, Li J, Cui X and He X wrote the manuscript. Gao W, Ou T and Jia J designed the research. Gao W and Li X analyzed the data. All authors read and approved the manuscript.

## Figures and Tables

**Table 1:** Assessment scores of time spent practicing with the training model of paracentetic suprapubic cystostomy (objective) for a sample case.

		Weight of the score for each step	Standard time for a perfect score (s)	Student A in the experimental group	
				Actual time (s)	Score
Experimental group	disinfection and draping	20	100	106	19
	local anesthesia and site selection for the suprapubic incision	5	20	39	3
	incision (skin+subcutaneous+front sheath)	10	20	9	11
	puncture (entered 1 cm farther after the sense of a breakthrough), check the urine outflow	20	30	34	18
	catheter placement (preset in advance)	10	25	26	10
	filling water in the bladder	5	10	9	5
	extubation of the catheter with the needle	5	15	16	5
	check the urine drainage	5	15	18	5
	two sutures on the skin surface and catheter fixation	10	70	226	7
	check the completeness and tightness of the bladder model	10	15	28	8
Total		100	320	511	91

**Table 2:** Assessment scores by urology experts of practice with the training model of paracentetic suprapubic cystostomy (subjective) for a sample case.

		Weight of the score for each step	Student A in the experimental group
			Score
Experimental group	disinfection and draping	20	18
	local anesthesia and site selection for the suprapubic incision	5	5
	incision (skin+subcutaneous+front sheath)	10	9
	puncture (entered 1 cm farther after the sense of a breakthrough), check the urine outflow	20	19
	catheter placement (preset in advance)	10	9
	filling water in the bladder	5	5
	extubation of the catheter with the needle	5	5
	check the urine drainage	5	5
	two sutures on the skin surface and catheter fixation	10	9
	check the completeness and tightness of the bladder model	10	10
Total		100	93

**Table 3:** Raw performance scores of all medical students by group and gender.

ID	Group	Gender	Performance final score	Performance level
1	Case	Female	92.2	Excellent
2	Case	Male	93.2	Excellent
3	Case	Female	83.6	Poor
4	Case	Female	90.8	Excellent
5	Case	Female	91.4	Excellent
6	Case	Female	86.6	Good
7	Case	Female	87.8	Good
8	Case	Female	93.2	Excellent
9	Case	Female	91.8	Excellent
10	Case	Female	92.8	Excellent
11	Case	Female	93.2	Excellent
12	Case	Female	91.2	Excellent
13	Case	Female	89.0	Good
14	Case	Female	85.2	Good
15	Case	Male	89.6	Good
16	Case	Male	91.6	Excellent
17	Case	Male	89.6	Good
18	Case	Male	89.8	Good
19	Case	Female	93.2	Excellent
20	Case	Male	89.8	Good
21	Control	Female	85.8	Good
22	Control	Female	68.4	Poor
23	Control	Female	86.4	Good
24	Control	Male	87.4	Good
25	Control	Male	82.0	Poor
26	Control	Female	77.2	Poor
27	Control	Female	87.2	Good
28	Control	Male	85.0	Good
29	Control	Female	91.4	Excellent
30	Control	Female	86.4	Good
31	Control	Male	88.8	Good
32	Control	Male	84.0	Poor
33	Control	Female	92.4	Excellent
34	Control	Female	92.0	Excellent
35	Control	Female	92.6	Excellent
36	Control	Female	87.6	Good
37	Control	Female	89.0	Good
38	Control	Female	88.6	Good
39	Control	Female	79.2	Poor
40	Control	Male	84.8	Poor

**Table 4:** Comparisons of final scores between the experimental group and control group.

	Experimental group	Control group	*p*-value
N	20	20	
Total score	91.0 (89.2-92.7)	86.8 (84.2-88.95)	0.002*
Performance level^a^			0.035*
Poor	1 (5%)	6 (30%)	
Good	8 (40%)	10 (50%)	
Excellent	11 (55%)	4 (20%)	

*p<0.05, represents significant differences between the experimental group and control group.

^a^Performance level was graded by total score; Poor: total score <85, Good: 85-90, Excellent: total score≥90.
